# Association between out-of-pocket health expenditure and the disease burden of diabetes mellitus: insights from GBD 2021

**DOI:** 10.3389/fpubh.2025.1601112

**Published:** 2025-07-25

**Authors:** Luyou Dong, Zhifu Dong

**Affiliations:** ^1^City University of Hong Kong, Kowloon, Hong Kong SAR, China; ^2^Chongqing Medical and Pharmaceutical College, Chongqing, China

**Keywords:** out-of-pocket expenditure, diabetes mellitus, disease burden, DALYs, GBD 2021

## Abstract

**Objectives:**

As one of the most common chronic diseases, diabetes mellitus poses a significant challenge to healthcare systems. This study analyzes the relationship between out-of-pocket (OOP) expenditure levels and the disease burden of diabetes mellitus, provides evidence-based recommendations for optimizing OOP expenditure strategies, and seeks to uncover any potential impact of healthcare inequalities on the disease burden of diabetes mellitus.

**Methods:**

This cross-sectional study was performed among 36 countries with varying percentages of OOP payments from *Health System in Transition*. Data on Disability-Adjusted Life Years (DALYs), obesity rates, OOP expenditure as a percentage of current health expenditure (CHE), and urbanization levels were sourced from the Global Burden of Disease (GBD) database, World Health Organization, and World Bank. Statistical analyses in RStudio included the Welch's two-sample *t*-test and multiple linear regression.

**Results:**

High OOP expenditure countries exhibited significantly higher diabetes-related DALYs (*M* = 965.98) versus low OOP groups (*M* = 556.33, 95% CI [103.99–715.32], *p* = 0.01). Regression analysis identified that low OOP expenditure, higher obesity rates, and greater urbanization levels were significantly associated with diabetes-related DALYs (β = −419.67, β = 37.31, and β = 8.07, respectively; all *p* < 0.05), explaining 51% of the variance (*R*^2^ = 0.51) with no evidence of multicollinearity (VIF <2).

**Conclusions:**

This study shows that countries with high OOP expenditure tend to experience a significantly greater disease burden of diabetes mellitus, with obesity and urbanization levels being important correlates of diabetes-related DALYs.

## 1 Introduction

Diabetes mellitus (DM) is a chronic metabolic disorder characterized by elevated blood sugar levels due to the pancreas's inability to produce sufficient insulin or the body's inability to effectively utilize the insulin produced (1). Globally recognized as a critical public health concern, diabetes affects millions worldwide, significantly impacting individuals' quality of life and posing substantial challenges to public health infrastructures. According to the International Diabetes Federation, over 536 million adults aged between 20 and 79 years were living with diabetes in 2021, with projections indicating an increase to 783 million by 2045 (2). Such growth in prevalence is anticipated to intensify the strain on healthcare systems globally, creating substantial economic and social burdens (3, 4).

A key determinant in managing and mitigating the health and economic impacts of diabetes is the out-of-pocket (OOP) payments implemented within a country. OOP payments, together with government financing schemes and compulsory health insurance, constitute the three main schemes of health financing models, which play a key role in determining the availability, affordability, and quality of healthcare services for people (5).

Although numerous studies have explored the epidemiology and clinical management of diabetes, relatively limited attention has been directed toward understanding how different levels of OOP expenditure are associated with the overall disease burden. Given this critical research gap, this study aims to analyze the impact of different OOP expenditure levels on the disease burden associated with DM. By providing robust, evidence-based insights into the relationship between OOP expenditure and diabetes outcomes, this study aims to analyze the relationship between OOP expenditure levels and the disease burden of DM, provide evidence-based recommendations for optimizing OOP expenditure strategies, and uncover the potential impact of health financing inequalities on the disease burden of DM.

## 2 Materials and methods

This cross-sectional study adopted a quantitative method to assess the relationship between OOP expenditure levels and the disease burden of DM.

Disability-adjusted life years (DALYs) mean the loss of 1 year of healthy life, which are the sum of years of life lost (YLLs) and years lived with disability (YLDs) (6). It is commonly used to quantify the burden of disease at the population level, and the diabetes-related DALYs were used to quantify to burden of DM in this study.

OOP payments refer to costs that patients must pay directly when neither public nor private insurance fully covers the expenses for healthcare goods or services. These payments encompass cost-sharing and other out-of-pocket expenses incurred by households. Additionally, it is preferable to include estimates of informal payments made to healthcare providers (5).

### 2.1 Study design

The research was conducted using a random sample of countries for which health systems have been reported in the journal *Health System in Transition*. The sample size was calculated to be 36, with α = 0.05, *Cohen's d* = 0.55, 1 – β = 0.8, and several predictors equal to 3. The 49 countries reported in the journal *Health Systems in Transition* were coded 1 to 49, and 36 of them were selected by RStudio random sampling. The 36 countries were categorized into two groups based on whether the proportion of OOP expenditure exceeded 25% of each country's current health expenditure (CHE). As established benchmarks for OOP expenditure thresholds are lacking in the literature, the 25% cutoff was subjectively selected based on the distribution of our sample data. This threshold clearly distinguished between high and low OOP groups (mean OOP% significantly higher in the high group) and was chosen as a more generous value to sensitively capture potential impacts of OOP expenditure on DM. Additionally, the obesity rates, identified as a disease-related indicator, and urbanization levels, classified as an economic-related indicator, were selected as the potential impact indicators to analyze the impact of OOP expenditure level on the disease burden of diabetes through control variables.

### 2.2 Data source

DALYs of DM, serving as an indicator of disease burden, along with the percentage of high body-mass index as a potential impact factor, were obtained from the Global Burden of Disease (GBD) database for each country in 2021. OOP expenditure as a percentage of CHE for each country in 2021 was obtained from the World Health Organization database to categorize the sample. The urbanization rate for each country in 2021 was obtained from the World Bank database as a potential impact factor.

### 2.3 Data analysis

The data were compiled and entered into RStudio for statistical analysis, allowing for the extraction and interpretation of the information.

A map was utilized to illustrate the distribution of countries across two groups. A box plot was employed to compare the disease burden of diabetes among countries with different percentages of OOP payments. Normality tests and variance chi-square tests were conducted in advance. Depending on the results, either the Mann–Whitney *U*-test, Welch's *t*-test, or independent samples *t*-test was utilized to assess differences between the groups. Multiple Linear Regression was used to analyze the impact of OOP expenditure on the disease burden of diabetes through control variables.

For all tests, values of *p* ≤ 0.05 were considered statistically significant.

## 3 Results

**Figure 1:**
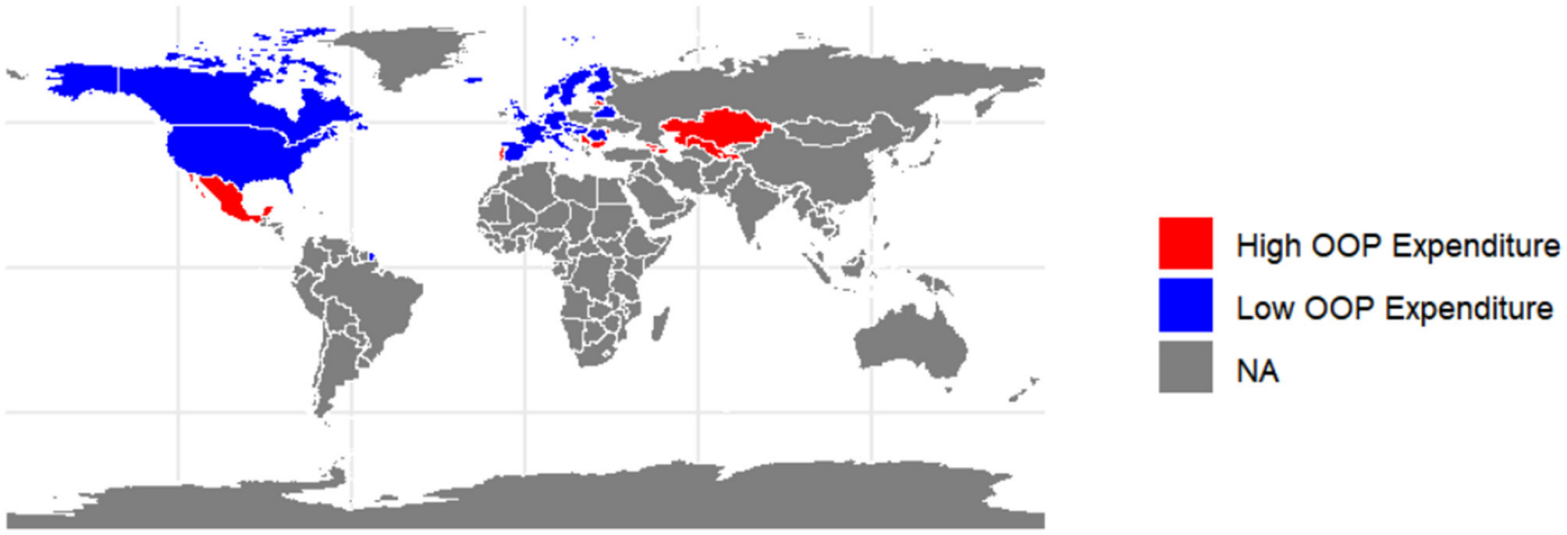
Distribution map of sampling countries by OOP expenditure level (36 countries, 2025).

The 36 countries included in the study were categorized into 12 high OOP expenditure countries and 24 low OOP expenditure countries (Table 1). These countries mainly cover Europe, Asia, and North America, with their distribution illustrated in Figure 1. The disease burden of DM, obesity rates, and urbanization levels according to this classification are presented in Table 2. Among a total of 36 countries, 12 (33.33%) are classified as having high OOP expenditure, and 24 (66.67%) are classified as having low OOP expenditure. The mean values for high and low OOP expenditure countries are 965.98 and 556.33 years, respectively. High OOP expenditure is also associated with a lower urbanization rate mean (58.96), compared to 78.12 for low OOP expenditure countries. The obesity percentages mean is 58.88 and 55.01 for high and low OOP expenditure countries, showing no significant difference.

**Table 1:** The list of high OOP expenditure and low OOP expenditure countries (36 countries, 2025).

**High OOP expenditure countries**	**Low OOP expenditure countries**
Tajikistan	United States
Armenia	Germany
Azerbaijan	United Kingdom
Uzbekistan	France
Portugal	Canada
Mexico	Switzerland
Moldova	Netherlands
Latvia	Slovenia
Serbia	Iceland
Bulgaria	Slovakia
Georgia	Norway
Kazakhstan	Romania
	Italy
	Denmark
	Estonia
	Sweden
	Belgium
	Finland
	Austria
	Spain
	Israel
	Belarus
	Hungary
	Czech Republic

**Table 2:** Diabetes-related DALYs, obesity percentages, and urbanization rates by OOP expenditure level (36 countries, 2025).

**Group**	***n* (%)**	**Diabetes-related DALYs (years) [Mean (SD)]**	**Obesity percentages [Mean (SD)]**	**Urbanization rates [Mean (SD)]**
High OOP expenditure	12 (33.33%)	965.98 (474.81)	58.88 (2.12)	58.96 (14.34)
Low OOP expenditure	24 (66.67%)	556.33 (149.59)	55.01 (4.53)	78.12 (12.65)

**Figure 2:**
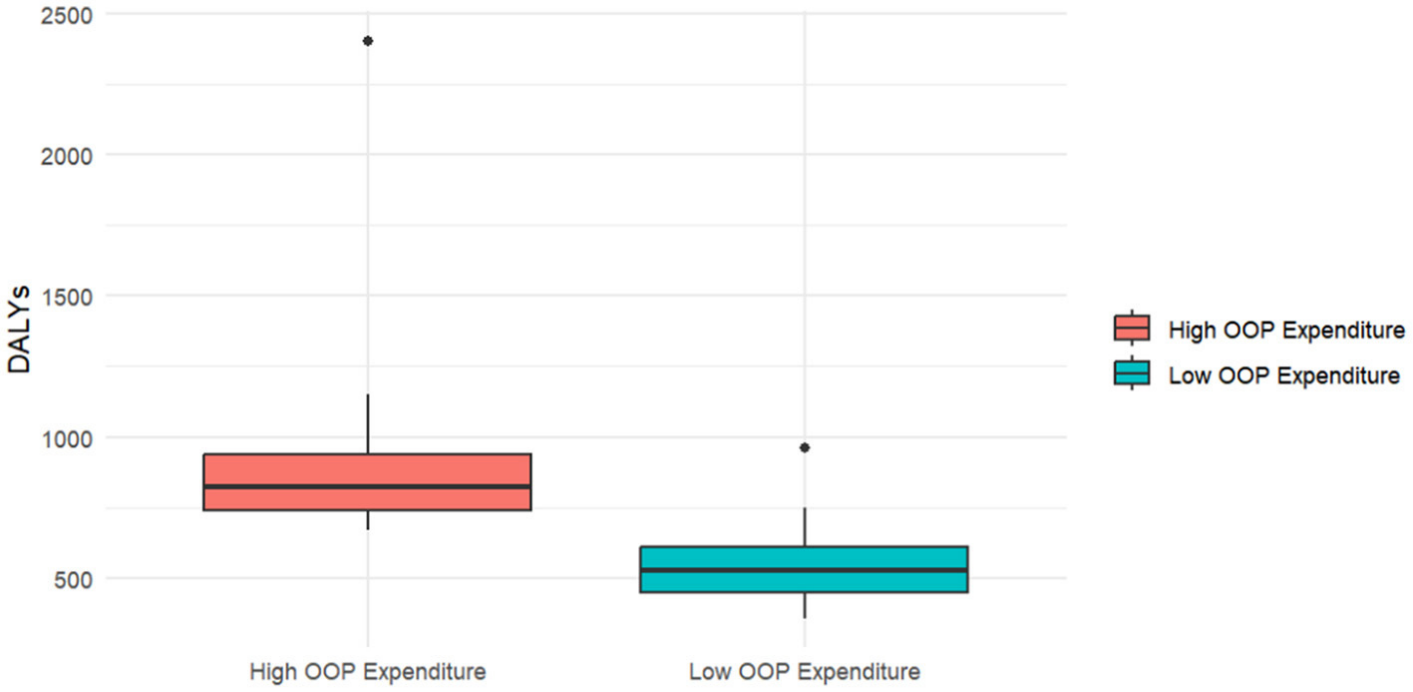
Diabetes-related DALYs by OOP expenditure level (36 countries, 2025).

Figure 2 displays the comparative distributions between these cohorts in Figure 2. There is a clear difference between the Diabetes-related DALYs of people with high and low OOP expenditure.

A Welch's two-sample *t*-test revealed a statistically significant difference in diabetes-related DALYs between high and low OOP expenditure groups [*t*_(12.11)_ = 2.92, *p* = 0.01]. The mean diabetes-related DALYs measured for the high OOP expenditure group (*M* = 965.98) were significantly higher than those for the low OOP expenditure group (*M* = 556.33), with a mean difference of 409.65 (95% CI [103.99, 715.32]), as shown in Table 3.

**Table 3:** Welch's two-sample *t*-test results comparing diabetes-related DALYs between high and low OOP expenditure groups (36 countries, 2025).

**Group**	**Diabetes-related DALYs (years) Mean**	**SD**	***n***	***t***	**d*f***	***p***	**95% CI**
OOP expenditure				2.92	12.11	0.01^*^	103.99, 715.32
High OOP Expenditure	965.98	474.81	12				
Low OOP Expenditure	556.33	149.59	24				

^*^p < 0.05, ^**^p < 0.01, ^***^p < 0.001.

A multiple linear regression model was fitted to examine the association between diabetes-related DALYs and OOP expenditure level, obesity rates, and urbanization levels. The model explained 51.4% of the variance in diabetes-related DALYs (*R*^2^ = 0.51, adjusted *R*^2^ = 0.469), with significant contributions from the low OOP expenditure model (β = −419.67, *p* < 0.001), obesity rates (β = 37.31, *p* < 0.001), and urbanization levels (β = 8.07, *p* = 0.02). Variance Inflation Factor (VIF) values for all predictors were below 2.00 (range: 1.29–1.61), indicating no substantial multicollinearity (Table 4).

**Table 4:** Multiple linear regression results for diabetes-related DALYs (36 countries, 2025).

**Predictor**	**β**	***t***	***p***	**VIF**
(Intercept)	−1,706.55	−2.28	0.03^*^	
Low OOP Expenditure Model	−419.67	−3.65	0.00^***^	1.61
Obesity rates	37.31	3.24	0.00^***^	1.29
Urbanization levels	8.07	2.37	0.02^*^	1.56

R^2^ = 0.51, Adjusted R^2^ = 0.47.

F_(3, 32)_ = 11.29, p = 3.28 × 10^−5^.

^*^p < 0.05, ^**^p < 0.01, ^***^p < 0.001.

VIF, Variance Inflation Factor; values <5 indicate negligible multicollinearity.

## 4 Discussion

The results suggest a strong association between the OOP expenditure level and the disease burden of DM, with countries having high OOP expenditures showing substantially greater diabetes-related DALYs compared to those with low OOP expenditures. The findings reveal a disparity of 409.65 years in mean diabetes-related DALYs between the two groups (*p* = 0.01), highlighting the critical role of OOP expenditure in the management of DM. This differential persists even when controlling for obesity rates and urbanization levels, as evidenced by a multiple regression model that explains 51.4% of the variance in diabetes-related DALYs (*F* = 11.29, *p* < 0.001).

The inverse relationship between OOP expenditure level and population health outcomes may be the result of financial barriers to accessing care due to high OOP expenditure (7, 8). The financial burden associated with high OOP expenditure may hinder adherence to treatment, regular blood sugar monitoring, and the utilization of preventive care for individuals with DM, potentially leading to delayed diagnoses and poorer outcomes related to DM management, which in turn exacerbates the overall disease burden of DM (9–11). The regression coefficients further quantify this relationship: countries with low OOP expenditure were associated with 419.67 fewer diabetes-related DALYs (β = −419.67, *p* < 0.001), highlighting a strong inverse association between patient cost-sharing and diabetes burden. While the cross-sectional design limits causal interpretation, the findings underscore a potentially important link between financial protection and public health outcomes.

Notably, obesity rates showed the strongest association among modifiable risk factors (β = 37.31, *p* < 0.001), underscoring the relevance of both fiscal policy considerations and population-level efforts to improve metabolic health (12, 13). The paradoxical association between urbanization and increased diabetes-related DALYs (β = 8.07, *p* = 0.02) warrants careful interpretation. While urban environments typically provide greater healthcare access, they may promote sedentary lifestyles and energy-dense dietary patterns (14). This dichotomy highlights the need for urban planning policies that integrate health-promoting infrastructure alongside the expansion of medical services.

To address the complex link between urbanization and diabetes burden, urban planning policies should integrate health-promoting infrastructure through actionable strategies (15). Multi-sectoral collaboration is needed to mitigate the impact of urbanization on diabetes outcomes, supported by evidence-based interventions such as transport mode choices and food environment regulations. Prioritizing pedestrian-friendly neighborhoods and cycling networks, enhancing non-motorized travel through traffic calming interventions, setting quotas for green spaces near residential areas, and developing multi-purpose recreational areas to encourage physical activity are interventions that can work together to create healthier and more sustainable compact cities (16). Dietary environmental legislation can bring health benefits, such as sugar taxes and labeling of processed foods (12, 17).

Geographic disparities in sampling distribution present both limitations and opportunities for future research. The overrepresentation of European nations in the low OOP cohort, compared to the predominance of Asian countries in high OOP groups, may introduce regional confounding factors. Cultural variations in dietary practices, physical activity norms, and healthcare-seeking behaviors could partially mediate the observed effects (18, 19). Nevertheless, the robust statistical controls (VIF <2.00) and comparatively large effect sizes strengthen confidence in the primary findings.

Several policy implications emerge from these results. The findings highlight the potential importance of reducing OOP expenditure as part of broader efforts to address the diabetes burden. These findings may inform discussions on how policymakers can improve accessibility to disease management by expanding health insurance coverage, increasing government financial investments in areas such as drug subsidies, primary healthcare services, and reducing OOP expenditure for patients. Considering obesity and urbanization factors, the implementation of multisectoral health interventions also deserves attention. OOP expenditure strategies should align with public health actions, such as allocating insurance funds to obesity screening, community nutrition programs, and health education; incorporating health-promoting urban designs and offering tax incentives for corporate wellness initiatives; enhancing rural healthcare infrastructure to address resource disparities. Additionally, regional collaboration and knowledge transfer, such as establishing platforms for DM prevention and cross-regional cooperation, can provide technical and financial support to high OOP expenditure countries. This approach can facilitate the development of transnational DM surveillance networks, thereby helping to reduce the disease burden of DM to some extent.

The study has several limitations. Firstly, the sample included only 36 countries, all located in the Northern Hemisphere, limiting the global generalizability of the findings. The absence of data from the Southern Hemisphere, such as Africa, South America, and Oceania, may introduce biases related to OOP expenditure level, disease burden, and other factors. OOP expenditure level and disease burdens in the Southern Hemisphere may differ significantly due to unique economic, cultural, and environmental factors. Additionally, rapid urbanization in some Southern Hemisphere countries may present distinct challenges, such as inadequate healthcare access and the coexistence of traditional and modern health practices. These factors could affect the impact of OOP expenditure level on diabetes burden. The cross-sectional design also precludes establishing causality between OOP expenditure level and diabetes burden, and the model explained only 51.4% of the variance in diabetes-related DALYs, suggesting potential omission of critical variables. Furthermore, the 25% OOP expenditure threshold was determined by our sample distribution rather than international standards. This subjective classification may limit the generalizability and comparability of findings across diverse healthcare contexts, particularly given the substantial heterogeneity observed among high OOP expenditure countries.

To conclude, this study finds that countries with higher OOP expenditure tend to experience a significantly greater burden of DM, with obesity rates and urbanization levels collectively associated with differences in diabetes-related DALYs. While causal interpretations cannot be made due to the cross-sectional design, these findings may inform future research and policy discussions.

## Data Availability

The datasets presented in this study can be found in online repositories. The names of the repository/repositories and accession number(s) can be found below: https://vizhub.healthdata.org/gbd-results/https://www.who.int/data/gho/data/indicators/indicator-details/GHO/out-of-pocket-expenditure-as-percentage-of-current-health-expenditure-(che)-(-)https://population.un.org/wup/downloads?tab=Urban%20and%20Rural%20Populations.
